# Functional Expression of Human Adenine Nucleotide Translocase 4 in *Saccharomyces Cerevisiae*

**DOI:** 10.1371/journal.pone.0019250

**Published:** 2011-04-21

**Authors:** Takashi Hamazaki, Wai-Yee Leung, Brian D. Cain, David A. Ostrov, Peter E. Thorsness, Naohiro Terada

**Affiliations:** 1 Department of Pathology, College of Medicine, University of Florida, Gainesville, Florida, United States of America; 2 Department of Biochemistry, University of Florida, Gainesville, Florida, United States of America; 3 Department of Molecular Biology, College of Agriculture, University of Wyoming, Laramie, Wyoming, United States of America; University of Groningen, Netherlands

## Abstract

The adenine nucleotide translocase (ANT) mediates the exchange of ADP and ATP across the inner mitochondrial membrane. The human genome encodes multiple ANT isoforms that are expressed in a tissue-specific manner. Recently a novel germ cell-specific member of the ANT family, ANT4 (SLC25A31) was identified. Although it is known that targeted depletion of ANT4 in mice resulted in male infertility, the functional biochemical differences between ANT4 and other somatic ANT isoforms remain undetermined. To gain insight into ANT4, we expressed human ANT4 (hANT4) in yeast mitochondria. Unlike the somatic ANT proteins, expression of hANT4 failed to complement an *AAC*-deficient yeast strain for growth on media requiring mitochondrial respiration. Moreover, overexpression of hANT4 from a multi-copy plasmid interfered with optimal yeast growth. However, mutation of specific amino acids of hANT4 improved yeast mitochondrial expression and supported growth of the *AAC*-deficient yeast on non-fermentable carbon sources. The mutations affected amino acids predicted to interact with phospholipids, suggesting the importance of lipid interactions for function of this protein. Each mutant hANT4 and the somatic hANTs exhibited similar ADP/ATP exchange kinetics. These data define common and distinct biochemical characteristics of ANT4 in comparison to ANT1, 2 and 3 providing a basis for study of its unique adaptation to germ cells.

## Introduction

The adenine nucleotide translocase (ANT) mediates the exchange of ADP and ATP across the inner mitochondrial membrane. Therefore, proper function of ANT is essential for the transfer of ATP synthesized in mitochondria to the cytoplasm. Most eukaryotes from yeast to humans have multiple ANT isoforms [1]. Unicellular organisms utilize different ANT isoforms depending on the availability of external nutrients and aeration. Multicellular organisms, express different ANT isoforms in a tissue-specific manner that are apparently adapted to the unique metabolic demands of the various tissues.

Recently, we and others identified a novel member of the ANT family, ANT4 (SLC25A31, AAC4, SFEC) in both humans and mice [2], [3], [4]. ANT4 is evolutionarily conserved in mammals and exclusively expressed in male germ cells of adult animals [5]. Although the previous gene knock-out study in mouse revealed that ANT4 was essential for the process of male germ cell meiosis in mice [6], neither the function of ANT4 in male germ cells nor the reason why ANT4 exists only within a limited spectrum of species is known.

The human ANT4 gene (hANT4) is predicted to encode a 315 amino acid protein. The protein contains the characteristic amino acid sequence (RRRMMM) shared by all known ADP/ATP carriers. hANT4 was demonstrated to possesses bona fide ADP/ATP transport upon reconstitution and assay of recombinant protein from *E. coli* into proteo-liposomes [3]. The reported kinetics of hANT4 were distinct from previously reported kinetics of other somatic hANTs, with comparatively lower affinity for adenine nucleotides and a higher *V*_max_. Unfortunately, the kinetics of ADP/ATP transport through hANT4 and somatic hANTs were not compared under comparable experimental conditions. Therefore, it is important to evaluate the differing biochemical characteristics of hANT4 and the somatic hANTs to elucidate the functional role of hANT4.

In order to determine the biochemical properties of the hANT4, we have chosen to heterologously express each hANT isoform in yeast and analyze their biochemical properties in parallel. Baker's yeast, *Saccharomyces cerevisiae* contains three paralogous genes encoding ADP/ATP carriers: *AAC1*, *AAC2* and *AAC3*. Yeast AACs have been extensively studied, taking advantage of the myriad molecular and genetic tools available in this organism [7]. The mitochondrial ADP/ATP exhange acitivity is not essential for cell growth under fermentation culture conditions and becomes essential only under non-fermentation conditions. This unique system provided the means to knock-out all three native AAC genes and insert heterologous hANT genes. The function of ADP/ATP exhange acitivity can be readily determined by following growth on non-fermentable carbon sources. Moreover, the hANT1, 2, 3 proteins have all been functionally expressed in yeast mitochondria [8], [9].

Here we expressed hANT4 protein in *AAC*- deficient yeast mitochondria along with somatic hANTs for parallel comparison. Using a similar methodology to that required for functional hANT1, 2 and 3 expression in yeast, hANT4 failed to complement the respiratory defect of yeast lacking the endogenous AAC genes. Moreover, overexpression of hANT4 led to deleterious effects on yeast cell growth. However, mutant forms of hANT4 protein were isolated that facilitated proper mitochondrial localization and complementation of *AAC*-deficient yeast. The ADP/ATP exchange kinetics of those modified hANT4 proteins compared favorably to the kinetics of the somatic hANTs expressed and analyzed under identical experimental conditions.

## Results

### Introduction of the human ANT gene into the AAC2 locus

We initially attempted to insert the full length hANT4 coding sequence into *AAC*-deficient yeast by homologous recombination at the *AAC2* locus. The idea was to select transformants based on their ability to grow on media requiring a functional mitochondrial respiratory system by providing sufficient adenine nucleotide transport activity to support growth of yeast on nonfermentable carbon sources as described previously [10]. A codon-optimized hANT4 ORF was amplified by PCR with primers that provided 50 bp of identity to the sequences immediately 5′ and 3′ of the *AAC2* start and stop codons. The amplified DNA was transformed into the *AAC*-deficient yeast strain bearing the KAN-MX6 genetic marker at the *AAC2* locus. All yeast strains in this study bear deletions of the *AAC1* and *AAC3* and are derived from strain TCY119 (Table 1). As controls, either *AAC2* or hANT2 sequences were similarly prepared by PCR and used to generate knock-ins at the same locus. Only the *AAC2* knock-in transformants appeared and grew on nonfermentable media (YPEG), suggesting that neither hANT2 nor hANT4 knocked-in at this locus supported respiratory growth. Previously, it was found that addition of the N-terminal sequence from yeast *AAC2* to the cognate position of bovine ANT1 significantly increased expression in yeast [11]. Therefore, we constructed chimeric hANT genes in which N-terminal sequences were replaced with the *AAC2* N-terminal 25 amino acids (yNhANTs) and repeated the knock-in protocol. Using this methodology, the yNhANT2 knock-in transformant clones could be isolated, and these yeast grew on YPEG. In contrast, yNhANT4 knock-in transfomants again failed the selection protocol.

**Table 1:** Yeast strains used in this study.

Strain^a^	Genotype^b^
TCY119^c^	*MATα ura3–52 leu2–3, 112 trp1-Δ1 ade2 his3-Δ1::hisG aac1-Δ1::hisG aac2-Δ1::kanMX6 aac3-Δ1::hisG* [*ρ+, TRP1*]
URA-AAC	*MATα ura3–52 leu2–3, 112 trp1-Δ1 ade2 his3-Δ1::hisG aac1-Δ1::hisG aac2-Δ1::URA3 aac3-Δ1::hisG* [*ρ+, TRP1*]
yAAC2	*MATα ura3–52 leu2–3, 112 trp1-Δ1 ade2 his3-Δ1::hisG aac1-Δ1::hisG aac2-Δ1::6xHis-AAC2 aac3-Δ1::hisG* [*ρ+, TRP1*]
yNhANT1	*MATα ura3–52 leu2–3, 112 trp1-Δ1 ade2 his3-Δ1::hisG aac1-Δ1::hisG aac2-Δ1::6xHis-yNhANT1 aac3-Δ1::hisG* [*ρ+, TRP1*]
yNhANT2	*MATα ura3–52 leu2–3, 112 trp1-Δ1 ade2 his3-Δ1::hisG aac1-Δ1::hisG aac2-Δ1::6xHis-yNhANT2 aac3-Δ1::hisG* [*ρ+, TRP1*]
yNhANT3	*MATα ura3–52 leu2–3, 112 trp1-Δ1 ade2 his3-Δ1::hisG aac1-Δ1::hisG aac2-Δ1::6xHis-yNhANT3 aac3-Δ1::hisG* [*ρ+, TRP1*]
yNhANT4	*MATα ura3–52 leu2–3, 112 trp1-Δ1 ade2 his3-Δ1::hisG aac1-Δ1::hisG aac2-Δ1::6xHis-yNhANT4 aac3-Δ1::hisG* [*ρ+, TRP1*]
yNhANT4 A30V	*MATα ura3–52 leu2–3, 112 trp1-Δ1 ade2 his3-Δ1::hisG aac1-Δ1::hisG aac2-Δ1::6xHis-yNhANT4 (A30V) aac3-Δ1::hisG* [*ρ+, TRP1*]
yNhANT4 P95S	*MATα ura3–52 leu2–3, 112 trp1-Δ1 ade2 his3-Δ1::hisG aac1-Δ1::hisG aac2-Δ1::6xHis-yNhANT4 (P95S) aac3-Δ1::hisG* [*ρ+, TRP1*]
yNhANT4 P95L	*MATα ura3–52 leu2–3, 112 trp1-Δ1 ade2 his3-Δ1::hisG aac1-Δ1::hisG aac2-Δ1::6xHis-yNhANT4 (P95S) aac3-Δ1::hisG* [*ρ+, TRP1*]
yNhANT4 S202L	*MATα ura3–52 leu2–3, 112 trp1-Δ1 ade2 his3-Δ1::hisG aac1-Δ1::hisG aac2-Δ1::6xHis-yNhANT4 (S202L) aac3-Δ1::hisG* [*ρ+, TRP1*]
yNhANT4 V5	*MATα ura3–52 leu2–3, 112 trp1-Δ1 ade2 his3-Δ1::hisG aac1-Δ1::hisG aac2-Δ1::6xHis-yNhANT4-V5::KanR aac3-Δ1::hisG* [*ρ+, TRP1*]
yNhANT4 A30V V5	*MATα ura3–52 leu2–3, 112 trp1-Δ1 ade2 his3-Δ1::hisG aac1-Δ1::hisG aac2-Δ1::6xHis-yNhANT4-V5 (A30V)::KanR aac3-Δ1::hisG* [*ρ+, TRP1*]
yNhANT4 S202L V5	*MATα ura3–52 leu2–3, 112 trp1-Δ1 ade2 his3-Δ1::hisG aac1-Δ1::hisG aac2-Δ1::6xHis-yNhANT4-V5 (S202L)::KanR aac3-Δ1::hisG* [*ρ+, TRP1*]

a Unless indicated, all strains were created in this study.

b Mitochondrial genome is bracketed.

c Strain source: [10].

**Figure 1:**
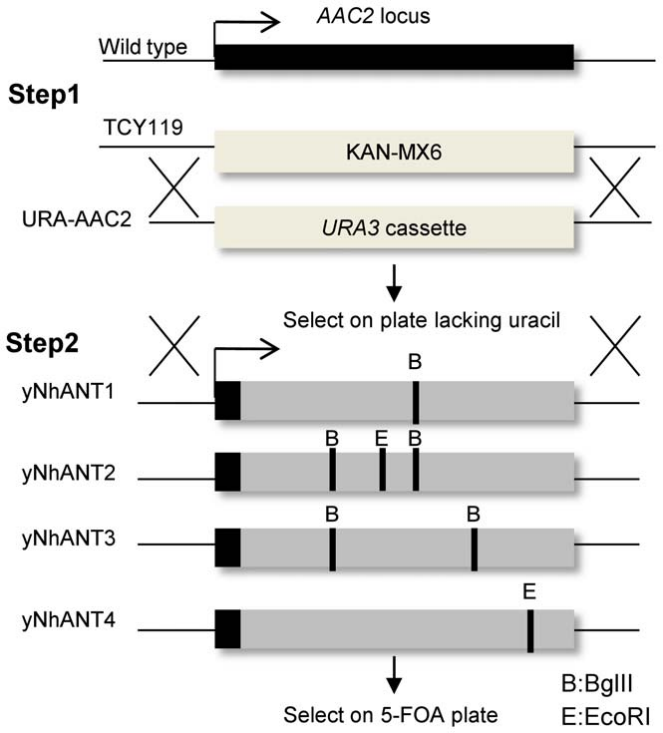
Strategy for introduction of hANTs into the AAC2 locus. Step1: KAN-MX6 cassette at the AAC2 locus in AAC triple mutant yeast (TCY119) was replaced with URA3 to establish the parent stain (URA-AAC2). Step2: PCR-generated N-terminal AAC + hANTs ORF fragments (yNhANTs) were used for transformation of URA-AAC2, and transformants were selected on rich glucose media containing 5-FOA.

**Figure 2:**
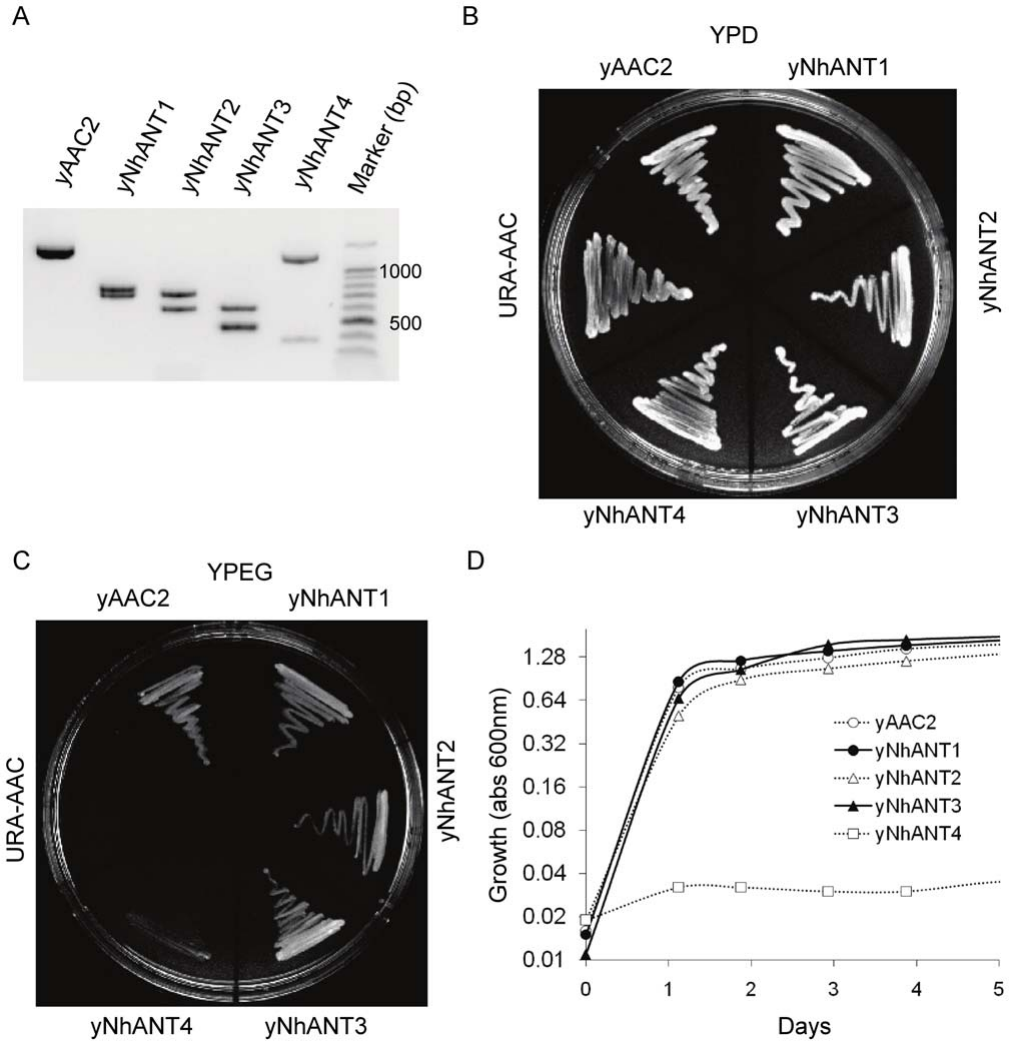
Characterization and growth of humanized-ANT yeast strains. (**A**) Confirmation of hANT gene insertion by PCR-restriction fragment length analysis. AAC2 locus was PCR amplified by primers positioned 5′ and 3′ of ORF. The fragments were digested with BglII and EcoRI, and separated on a 2% agarose gel. (**B**) Growth of hANT yeast on complete glucose medium (YPD). (**C**) Growth of hANT yeast on complete ethanol-glycerol medium (YPEG). (**D**) Growth curves of hANT yeast in YPEG determined by turbidity (O.D.600).

To insert the hANT4 gene into the yeast *AAC2* independent of its ability to complement the ATP/ADP exchange function, a two-step strategy was adopted (Fig. 1). First, the KAN-MX6 marker present at the *AAC2* locus of TCY119 was replaced with the common yeast selectable marker URA3 (“URA3-AAC2” in Fig. 1). In the second step, each hANT knock-in construct was transformed into the URA3-AAC2 strain to allow homologous recombination at URA3-AAC2 site. Transformants were identified by selecting for 5-FOA resistance on rich glucose media. Yeast lacking the URA3 gene are resistant to the cyotoxic effects of 5-FOA [12]. In this way, yeast strains carrying yNhANT4 gene at the *AAC2* locus were successfully isolated and propagated (yNhANT4). yNhANT1, 2, 3 and *AAC2* expressing strains were similarly generated (Fig. 2A). All yeast strains were capable of growth on fermentable carbon sources (YPD, rich-glucose media) (Fig. 2B). *AAC2* and yNhANT1, 2, 3 knock-in yeast displayed abundant growth on nonfermentation culture conditions (YPEG). However, yNhANT4 did not grow on YPEG when directly streaked from YPD grown cells (Fig. 2C). Even after incubation for over 1 week, yNhANT4 strain did not grow on YPEG (Fig. 2D). We concluded that modifying the N-terminus of hANT4 with *AAC2* N-terminal sequences was not sufficient to provide adequate translocator activity for growth of yeast on nonfermentable carbon sources.

### Effects of excessive expression of hANT4

**Figure 3:**
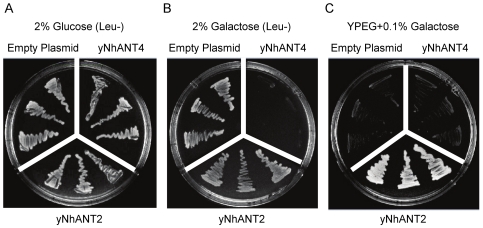
Over-production of yNhANT4 by high-copy plasmid inhibits yeast cell growth. Both yNhANT4 and yNhANT2 ORF were cloned into a high-copy plasmid and expressed under the control of the GAL1 promoter. Three independent isolates, each transformed with one of the indicated plasmids (Empty plasmid, yNhANT4, and yNhANT2) were streaked on medium as indicated. (**A**) glucose medium, (**B**) galactose medium, and (**C**) YPEG + 0.1% galactose.

We suspected that hANT4 expression from a single copy at the *AAC2* locus might be insufficient to compensate for the loss of endogenous ATP/ADP translocators. Therefore, hANT4 gene expression was increased by transforming yeast with a high-copy plasmid, pESC-Leu2d [13] bearing the yNhANT4 gene. This plasmid, pESC-Leu2d::yNhANT4, is maintained both at high copy and expresses the gene under control of the strong *Gal1* promoter. pESC-Leu2d::yNhANT2, a similarly constructed control plasmids with yNhANT2, or the empty vector were introduced into the triple *AAC* knock-out yeast (URA3-AAC2) (Fig. 3). The transformed yeast were incubated on glucose media lacking leucine (Fig. 3A), galactose media which induces expression of the respective ANT genes (Fig. 3B), and media containing a nonfermentable carbon source (Fig. 3C). When yNhANT4 expression was induced by addition of galactose, yeast cell growth was suppressed (Fig. 3B). Overexpression of yNhANT2 did not affect the growth on the fermentable carbon source galactose, and indeed yNhANT2 overexpression supported growth on nonfermentable carbon sources (Fig. 3C). These observations led us to conclude that the overexpression of yNhANT4 was unfavorable, and that further modification of yNhANT4 or the yeast genome would be required for functional reconstitution of yNhANT4 in yeast.

### EMS mutagenesis and functional screening of yNhANT4 yeast

**Figure 4:**
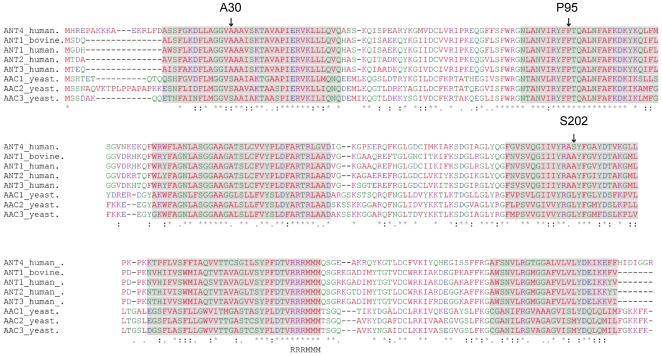
Primary amino acid sequence alignment of various ANTs. A30, P95 and S202 of hANT4 are indicated by arrow. Transmembrane domains are shown as shaded sequences.

The *AAC2* promoter is highly induced when yeast are transferred from fermentable to nonfermentable carbon sources [14]. Therefore, we hypothesized that while the lower yNhANT4 expression of glucose grown cells does not adversely affect cell growth, higher expression in YPEG may be inhibitory. To combat this putative intolerance to the heterologous protein, we isolated mutant forms of yNhANT4 that allowed yeast to grow on nonfermentable carbon sources. After treating yNhANT4 yeast strains with the mutagen EMS, four independent mutant strains were isolated that were capable of respiratory growth. The *AAC2*/yNhANT4 locus of each was subjected to DNA sequencing and each contained a point mutation in the yNhANT4 ORF that changed a single amino acid (Table 2). Alignment of the human ANT isoforms, yeast AAC isoforms and bovine ANT1 revealed that the hANT4 amino acid sequence is more than 70% identical to other mammalian ANT isoforms and more than 50% identical to the yeast AACs. The putative substrate binding sites as well as the signature RRRMMM motif are highly conserved between the isoforms and across species (Fig. 4). The three amino acid residues that were the sites of mutation in hANT4 that allowed complementation of *AAC*-deficient yeast varied in their degrees of conservation. A30 of hANT4 is conserved among mammals across all ANT isoforms but not conserved in yeast. P95 of hANT4 is conserved in all ANT related sequences. S202 of hANT4 is unique to ANT4 protein in all mammalian species examined (chimpanzee, cow, dog, mouse, rat and opossum) (data not shown).

**Table 2:** Summary of mutation sites that facilitate functional expression of yNhANT4 in yeast.

No.	Strain	Nucleotide change	Amino acid change	Corresponding position in	
				hANT 1, 2 and 3	*AAC2*
1	yNhANT A30V	GCT to GTT	A30V	A18	S33
2	yNhANT P95S	CCA to TCA	P95S	P83	P99
3	yNhANT P95L	CCA to CTA	P95L	P83	P99
4	yNhANT S202L	TCA to TTA	S202L	A190	L206

**Figure 5:**
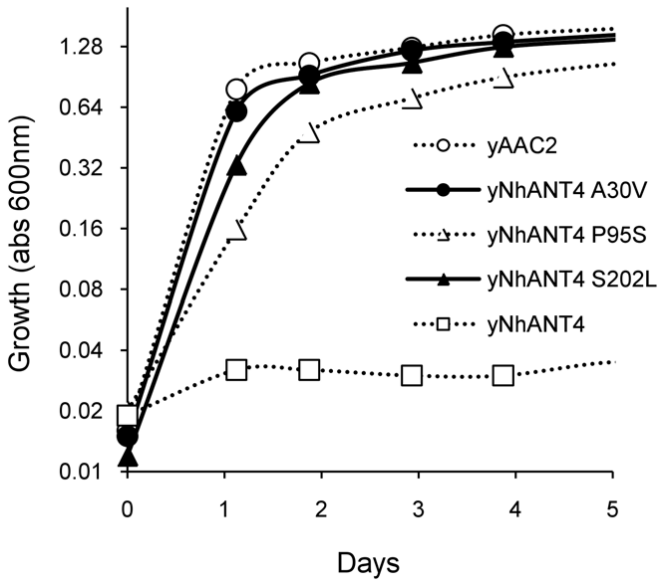
Growth of various yNhANT4 mutant yeasts on nonfermentable carbon sources. The hANT4 mutant alleles that complemented the aacΔ yeast were re-introduced into the parent yeast (URA-AAC2). The yeast strains were cultured in rich nonfermentable carbon source media (YPEG) at 30°C for the indicated time. Growth was monitored by turbidity (O.D.600).

We determined that the mutations in yNhANT4 were indeed responsible for the growth complementation on YPEG by reintroducing the mutated yNhANT4 alleles back into the parental strain. All the four alleles supported yeast growth on YPEG. The A30V mutation of yNhANT4 proved the strongest allele by showing rapid growth on nonfermentable carbon sources comparable to that of yeast bearing the wild-type yeast allele *AAC2* (Fig. 5). Although each amino acid mutation within yNhANT4 was sufficient to complement growth on nonfermentable carbon sources, the mutant yeasts that were originally recovered from EMS mutagenesis might have contained additional mutations outside of the yNhANT4 that also contributed to improved growth on nonfermentable carbon sources. To test this hypothesis, the mutant yNhANT4 locus for three of the isolates was first replaced by *URA3*, and then wild type yNhANT4 was reintroduced using 5-FOA selection as described above. None of the mutants bearing the non-mutated yNhANT4 allele could support growth on nonfermentable carbon sources. Therefore, we concluded that the A30V, P95S and S202L amino acid substitutions were sufficient for functional expression in yeast.

### Improved mitochondrial expression in hANT4 mutant strains

**Figure 6:**
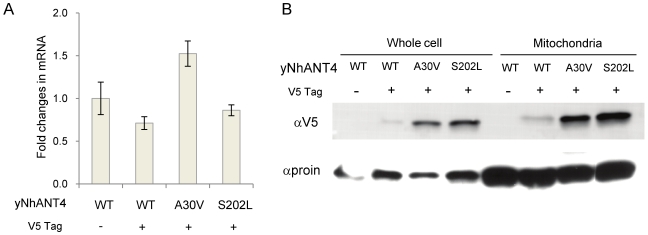
Mutations increase mitochondrial ANT4 protein levels. The V5-tag was introduced at the C-terminus of yNhANT4 in yeast strains as shown. All yeasts were cultured in rich glucose media (YPD). (**A**) hANT4 mRNA level determined by real time PCR. ALG9 expression level was used to normalize hANT4 expression. (**B**) hANT4 protein level in whole cells and isolated mitochondria was determined by western blotting using an anti-V5 epitope antibody. As a loading control, the membrane was immunostained with an anti-yeast porin antibody. WT: unmutated strains.

To further investigate the role of amino acid residues A30, P95 and S202 in yNhANT4, we analyzed yNhANT4 transcript and protein levels in isogenic parent and mutant yeast strains. The V5-tag was added to the C-terminus of yNhANT4 and mutant yNhANT4 genes to allow detection of yNhANT4 protein. The presence of the V5-tags did not alter the growth characteristics on YPD or YPEG in comparison to the parental untagged strains. The yNhANT4-V5 yeast strains were cultured using the same media, rich glucose-containing media (YPD). When the transcript levels of the yNhANT4 gene from each strain were compared by real-time PCR, there were no significant differences between the parent and mutants (Fig. 6A). However, western blot analysis using anti-V5 antibody on whole cells and isolated mitochondria revealed that the amount of yNhANT4 protein in mitochondria was lower in the native yNhANT4-V5 strains than the A30V and S202L mutant strains (Fig. 6B). We also confirmed that yNhANT4 protein expression levels in P95S and P95L strains were similar to those in A30V and S202L strains in western blot analysis using anti-His antibody (data not shown). Based upon these observations, the increased amount of yNhANT4 protein found in mitochondria of mutant strains is likely due to a post-transcriptional event.

### ADP/ATP exchange kinetics of hANT4

**Figure 7:**
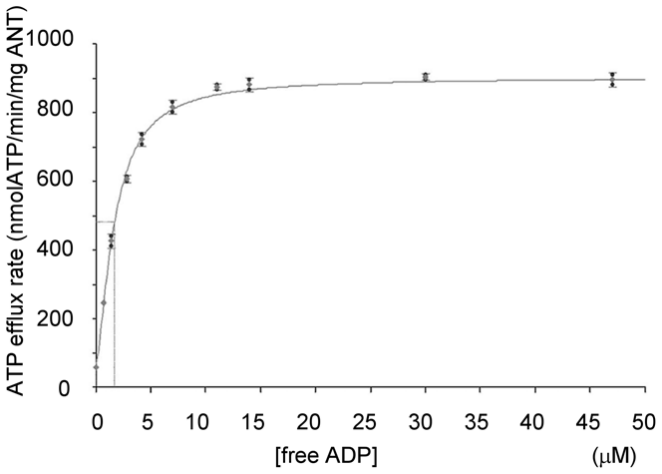
ADP/ATP exchange kinetics of yNhANT4 A30V in yeast mitochondria. The exchange reaction was initiated by adding various concentrations of ADP to freshly isolated mitochondria. The initial linear part of the kinetic curve was used as the initial velocity for the ATP efflux rate. The initial velocity was plotted against the substrate concentration [free ADP] using a 4-parameter logistic fit to describe the data shown here.

The ADP/ATP exchange kinetics of hANT4 was compared to the other somatic hANT isoforms. Mitochondria were prepared from hANT expressing yeast grown on nonfermentable carbon sources, and the efflux rate of ATP was measured as a function of changes in external ADP concentration. A representative hANT4 A30V kinetic curve is shown in Fig. 7 and the kinetic parameters obtained from each hANT are summarized in Table 3. All ATP/ADP translocators tested (hANT1, 2, 3, 4 and yeast AAC2) have a *K*_M_ for ADP in the micromolar range, consistent with previously reported values [8]. The hANT4 *K*_M_ values varied several fold depending on the particular allele of hANT4, with two of the three mutant proteins (A30V and S202L) showing lower *K*_M_ values for ADP than hANT1, 2 and 3. Interestingly, the difference found in the kinetic parameters of the mutant hANT4 proteins corresponded to the relative growth rates of yeast on nonfermentable carbon sources (**Figure 5**). The hANT4 bearing the A30V mutant form was fastest growing and has a low *K*_M_ and high *V*_max_. Mutant hANT4 proteins that supported growth less well on nonfermentable carbon sources had either a greater *K*_M_ (P95S) or smaller *V*_max_ (S202L).

**Table 3:** ADP/ATP exchange kinetics of hANTs measured on isolated yeast mitochondria.

Strain	*K*_M_	*V*_max_
	µM [free ADP]	nmolATP/min/mg ANT
yAAC2	1.1	1430
yNhAnt1	2.7	1190
yNhAnt2	5.5	1640
yNhAnt3	7.6	1300
yNhAnt4 A30V	1.9	950
yNhAnt4 P95S	10	1650
yNhAnt4 S202L	0.96	270

The given values are the average of at least two independent experiments. The standard error values were less than 10% for *K*_M_ and 20% for *V*_max._

## Discussion

*Saccharomyces cerevisiae* has proven to be a useful experimental system for investigating the fundamental biochemical properties of ADP/ATP exchange across the inner mitochondrial membrane [7]. Since mitochondrial ADP/ATP exhange acitivity in yeast is not essential during fermentative growth but is essential for growth using nonfermentable carbon sources, it was possible to introduce and biochemically analyze nonfunctional or sub-functional ANTs. Consequently, we were able to knock-out all three native *AAC* genes and insert heterologous genes corresponding to hANT1, 2, 3, and 4 at the yeast *AAC2* locus.

There are some limitations of this heterologous expression method. Several previous reports as well as data presented here demonstrated that the N-terminal sequence greatly influences the functional localization of ANT proteins [15]. Apparently the mammalian ANT proteins lack a necessary signal for compatibility with the yeast mitochondrial inner membrane protein transport machinery. Interestingly, hANT4 required additional mutations for functional expression in yeast mitochondria. These mutations were all missense mutations affecting A30, P95 or S202 in hANT4 protein and improved yeast mitochondrial expression. Without these modifications, hANT4 protein was unstable in yeast (Fig. 6). Accumulation of excess unfolded protein could be the reason that over-production of the yNANT4 protein suppressed the yeast cell growth (Fig. 3).

**Figure 8:**
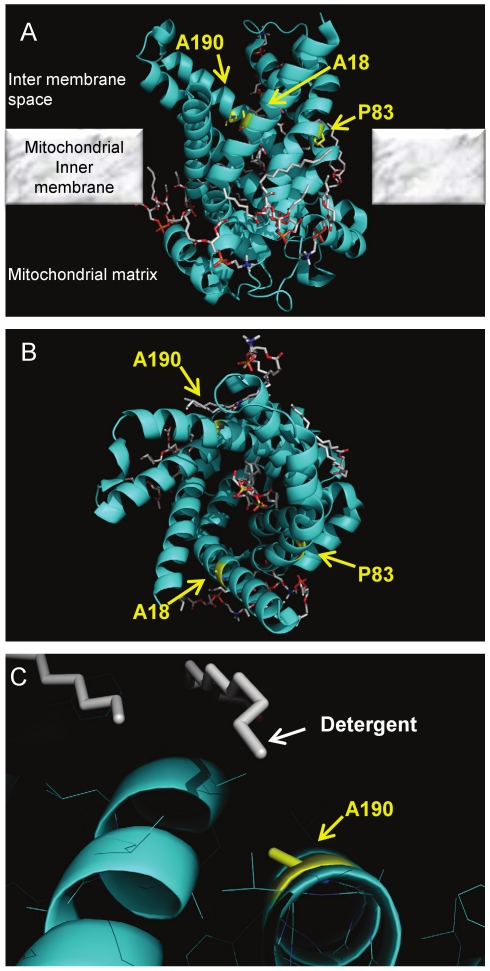
Location of hANT4 mutation sites in the bovine ANT1 structure. Based on primary amino acid sequence alignment (Fig. 4), A30, P95 and S202 of hANT4 correspond to A18, P83 and A190 of bovine ANT1, respectively. Those sites are highlighted in yellow on the bovine ANT1 structure (Protein Data Bank ID: 1OKC). The ANT1 crystal structure is oriented to show the inter membrane space at the top and the mitochondrial matrix at the bottom (**A**). Panel **B** is rotated 90 degrees about a horizontal axis in the plane of the page compared to panel **A** to show the ANT1 region facing the cytoplasm. Panel **C** shows how fatty acid detergent elements are in close proximity to a specific position in the ANT1 crystal structure (S202 in ANT4, A190 in ANT1).

It remained unclear how the specific amino acid substitution at the residues (A30, P95 and S202) allowed functional expression in yeast mitochondria. Therefore, we mapped these amino acids onto a three-dimensional structure of hANT4, using the sequence alignments and the crystal structure of bovine ANT1 [16] as a guide (Fig. 4 and 8). Interestingly, all three mutation sites were located in transmembrane domains and are predicted to be located at similar levels with respect to the mitochondrial membrane. All three sites are oriented towards solvent, and two of the sites (P95S and S202L) are located in positions that may permit interaction with lipid since these residues are in close proximity to the LAPAO detergent in the crystal structure of ANT1 [16]. Indeed, both substitutions of A30V and S202L increase hydrophobicity of these sites. The substitution of P95S might impose flexibility to fit the yeast lipid environment. It should be noted that the substitution of P89L in *AAC1* that corresponds to the P95L substitution in hANT4 has shown to alter functionality of the protein in the native phospholipid environment of yeast [17]. A previous study demonstrated that modifications of amino acids of somatic hANT proteins near a putative cardiolipin interaction site improved yeast growth in reduced oxgen conditions, and suggested that differences of lipid composition between the mitochondrial inner membranes of yeast and mammals might limit proper function [18].

Since ANT4 is exclusively found in mammalian germ cells and sperm mitochondria, it is reasonable to speculate that hANT4 may have evolved to adapt to the lipid environment of those cell types. Interestingly, the relatively subtle amino acid changes isolated in hANT4 influenced the ADP/ATP exchange kinetics even though those changes occurred far from the substrate binding site (Table 3). This may also suggest that subtle changes in lipid composition of inner membrane may alter hANT function. Indeed, cardiolipin is a critical component for exchange activity in both yeast and mammalian ANT [19], [20]. Levels of phospholipids and cholesterol have also been shown to affect the exchange function of mammalian ANTs [21]. In the case of ANT4, oxidation or other damage to lipids could alter the exchange function of ANT4, and potentially affect male germ cell meiosis or sperm motility.

It is technically challenging to determine the ADP/ATP exchange kinetics of ANT proteins, which are influenced by various factors depending on the methodology. Therefore, the kinetic values vary considerably in literature [7]. The previously reported transport kinetics of hANT4 had higher *K*_M_ values, 72 µM for ADP and 120 µM for ATP in a liposome reconstitution system using purified hANT4 [3]. In the present study, we found that the *K*_M_ values were much lower in all three mutant hANT4 proteins that are compatible with those of somatic hANTs (Table 3). Although our hANT4 peptides contain mutations required for proper assembly and stability of the proteins in yeast mitochondrial membrane, these mutation sites are distant from the ADP/ATP binding pocket and unlikely to substantially change the substrate binding affinity *per se*. Indeed, the substitution of A18V in hANT2 protein, corresponding to A30V in hANT4, did not significantly change the kinetics of ADP/ATP in yeast mitochondria (data not shown). To this end, our data suggest that properly assembled hANT4 protein may have similar ADP/ATP exchange kinetics with those of the somatic hANTs.

In this study, native *AAC2* ORF was replaced with hANT sequences by homologous recombination. There is a distinct advantage to having a chromosomally borne transgene as an expression system as compared to a plasmid-based system. Using the experimental design described here, all cells in the culture contained the transgene and expressed it at the same level. In contrast, plasmid numbers vary from cell-to-cell with as many as 50% of cells in a culture under selection lacking the plasmid. This is particularly important for physiological studies of carbon source utilizations, studies that require transitions between growth conditions or media, and purification of proteins from large batch cultures. This yeast expression system can be used as a starting source to obtain structural information of hANT proteins. Additionally, these yeast strains will be a useful tool for high-throughput screening in drug discovery [22]. Small compounds identified in this way that specifically inhibit hANT4 function may have use as male contraceptives.

To date, there is no evidence of hANT4 gene mutations associated with a human disease. However, recent advances in sequencing technology have documented millions of novel SNP variants from large populations [23]. So far 18 hANT4 variants have been archived in the database, including 6 non-synonymous variants in the coding region. It will be interesting to see if any of those variants correlate with pathology. Association of any of these variants with a particular diseases must await further progress on genome-wide association studies and whole genome sequencing projects for specific diseases [24]. If a certain hANT4 variant is found to be associated with a human disease, functional consequences of the variant is readily testable using the expression system and techniques described here.

## Materials and Methods

### Strains and media

All *S. cerevisiae* strains were derived from D273-10B and the genotypes of strains used in this study are summarized in Table 1 [10]. Yeast strains were grown in a variety of media. Complete glucose medium (YPD) contained 2% glucose, 2% bacto peptone, 1% yeast extract, 40 mg/liter adenine, 40 mg/liter tryptophan. Complete ethanol-glycerol medium (YPEG) contained 3% glycerol, 3% ethanol, 2% bacto peptone, 1% yeast extract, 40 adenine, 40 mg/liter tryptophan. Synthetic dextrose medium (SD) was 2% glucose, 6.7 g/liter yeast nitrogen base without amino acids (Difco), supplemented with yeast synthetic drop-out media and/or appropriate amino acids, adenine, and uracil (Sigma). For solid media, bacto agar (Difco) was added at 18 g/liter. 5-fluoroorotic acid (5FOA) was added as appropriate at 1 g/liter (Zymo) or geneticin (G418) was added at 200 µg/ml.

### Insertion of URA3 gene into the AAC2 locus

Generation of triple null mutations of *AAC1*, *AAC2* and *AAC3* (TCY119) was previously described [10]. In TCY119, the *AAC2* locus was disrupted by a KAN-MX6 cassette imparting resistance to geneticin. We replaced the KAN-MX6 cassette with the *URA3* to allow knock-in hANT genes via homologous recombination using 5-FOA selection. To target the URA3 gene to the *AAC2* locus, a *URA3* cassette was generated containing DNA fragment flanked by sequence homologous to the ∼200 bp immediately up- and downstream of the *AAC2* ORF [25]. Briefly, *AAC2* ORF upstream and downstream sequences were amplified by PCR using primer set F1+R1 and F2+R2 separately. The *URA3* was amplified using primer set U1+U2, which contained the reverse complement sequence of either R1 or F2. Purified PCR fragments were annealed and amplified by the primer set F1+R2. The PCR-generated DNA fragments were used to transform TCY119 by the LiAc/SS Carrier DNA/PEG method [10]. Transformants were selected on SD plates lacking uracil. Insertion of *URA3* into the *AAC2* locus of the yeast (URA-AAC2) was verified by PCR and subsequent sequencing. Primer sequences denoted above are listed in supplemental Table S1.

### hANT1, 2, 3 and 4 knock-ins in the AAC2 locus

All hANT knock-in DNA fragments were constructed using the same PCR strategy described above. To amplify ORFs for hANT1, 2 and 3, human cDNAs were used as the PCR template. For the hANT4, a codon-optimized hANT4 ORF for expression in yeast was synthesized (MR. GENE GmbH) and used as a template. The hANT4 ORF sequences used in this study are shown in supplemental Figure S1. For making the 6X histidine tagged yeast *AAC2*, genomic DNA (TCY122) was amplified by PCR with primer sets F1+HisR and HisF+R2. Purified products were combined and further amplified using primer set F1+R2. The PCR products were transformed into URA-AAC2 followed by 5FOA selection to generate the His-tagged *AAC2* knock-in strain (yAAC2). For making the chimeric DNA fragment of containing the 5′His-AAC2 N-terminal with hANTs, genomic DNA from (yAAC2) was used as the PCR template and amplified with primer sets F1+yNR. Each hANT ORF was amplified with primers that contained the reverse complement sequence of yNR for the forward primer (yNRhANTs-F) and F2 as a reverse primer (F2ANTs-R). Three of the PCR products (HisAAC2 N-terminus with 5′ arm, each hANT ORF and F2+R2 fragment for 3′ arm) were combined and amplified using primer set F1+R2. The PCR generated knock-in constructs were transformed into *AAC* null yeast (URA-AAC2). Transformants were selected on 5-FOA plates. Insertion of the targeted DNA construct into *AAC2* was verified by PCR and the ORF of the locus was sequenced. Primer sequences denoted above are listed in supplemental Table S1.

### Plasmid constructions

pESC-Leu2d empty vector was obtained from Jay D. Keasling (U of California) (Addgene plasmid 20120) [26]. The yNhANT4 and yNhANT2 ORFs were PCR amplified with the following primers containing Xho1 and Nhe1 sites: forward Xho1yNF for both yNhANT4 and yNhANT2; reverse Nhe1hA4R for yNhANT4, Nhe1hA2R for yNhANT2. PCR fragments were cloned into pESC-Leu2d empty vector using the Xho1 and Nhe1 sites. Primer sequences denoted above are listed in supplemental Table S1.

### Ethyl methanesulfonate (EMS) mutagenesis

Yeast cells were washed with 0.1 M sodium phosphate (pH 7.0), and resuspended in 1.7 ml of this buffer supplemented with 50 µl EMS (Sigma). After one hour of incubation at 30°C, 50% of the cells were still alive, and mutagenesis was stopped by adding 8 ml of 5% w/v sodium thiosulfate. Cells were plated on YPEG media at a variety of dilutions and incubated at 30°C until colonies appeared.

### V5 tagging hANT4

The V5-tag epitope (GKPIPNPLLGLDST) [27] was introduced at the C-terminus of yNhANT4 by homologous recombination. First, the *ADH1* terminator sequence and KanR cassette were PCR amplified with a forward primer containing the V5-tag sequence (V5F) and a reverse primer containing the immediate downstream sequence of the *AAC2* ORF (V5R). Plasmid pKT0127 was used as a template DNA (kind gift of Kurt S. Thorn (Harvard U) (Addgene plasmid 8728)). To add the 5′ homologous arm, the PCR product was further amplified with a forward primer containing 40 bp of 3′ sequence of hANT4 ORF excluding stop codon (hANT4V5F). PCR generated DNA fragments were transformed into yNhANT4 yeast and each of the mutants. Transformants were selected by plating onto YPD containing G418. The V5-tagged hANT4 ORF in each clone was verified by sequencing. Primer sequences denoted above are listed in supplemental Table S1.

### Isolation of yeast mitochondria

Mitochondrial isolation was performed by standard protocols [28], [29]. Yeast strains were treated with zymolyase (Seikagaku America) to generate spheroplasts, and then broken with a Dounce homogenizer. Mitochondria were collected by differential centrifugation.

### Western blotting

Total protein was separated with sodium dodecyl sulfate–12% polyacrylamide gel electrophoresis and then transferred to a nitrocellulose membrane. The following were used as primary antibodies: anti V5-tag (Invitrogen), anti Porin (Invitrogen) and anti His-tag (Cell Signaling). Peroxidase-conjugated immunoglobulin G (Cell Signaling) was used as the secondary antibody followed by enhanced chemiluminescence (ECL) detection (Thermoscientific).

### Real-time PCR

Total RNA was isolated from spheroplasts using the RNA aqueous kit (Ambion) and treated with DNAase I using Turbo DNA free kit (Ambion). cDNA was synthesized using a high capacity cDNA archive kit (Applied Biosystems). Real time PCR was performed using SYBR green master mix (Applied Biosystems) with gene specific primers; for hANT4, primer set hANT4F and hANT4R and for ALG9, primer set ALG9F and ALG9R were used. ALG9 expression was used as reference gene to normalize hANT4 expression level [30]. Normalized expression was calculated by formula:{(Efficiency^hANT4^)^ CT hANT4^/ (Efficiency^ALG9^) ^CT ALG9^} using qGene software [31]. Primer sequences denoted above are listed in supplemental Table S1.

### ADP/ATP exchange assay in isolated mitochondria

The ADP/ATP exchange assays were performed essentially as described [8] with a slight modification. Briefly, freshly isolated mitochondria were added to reaction buffer (0.6 M mannitol, 0.1 mM EGTA, 2 mM MgCl2, 10 mM KPi, 5 mM α-ketoglutarate, 0.01 mM Ap5A, 10 mM Tris-HCl, pH 7.4) containing the ATP detection system (2.5 mM glucose, hexokinase (2 E.U.), glucose-6-phosphate dehydrogenase (2 E.U.), 0.2 mM NADP). The exchange reaction was initiated by adding various concentrations of ADP. The ATP efflux rate was monitored continually by monitoring the rate of NADPH formation (increase in absorbance at 340 nm). The initial linear part of the kinetic curve (first 3 minutes) was used to calculate the initial velocity, and the substrate concentrations [free ADP] were calculated using the program Win MAXC v2.51 created by Chris Patton (http://stanford.edu/~cpatton/). The reactions volume was 200 ul, and reactions were carried out in 96 well microtiter plates and read by Synergy HT plate reader (BioTek). Kinetic parameters were obtained by 4-parameter logistic fitting using Gen5 Data analysis software (BioTek). The amount of ANT protein in the reaction was quantified by western blotting using recombinant human His-tagged ANT proteins as a standard. The density of the blot was measured by using NIH ImageJ software (http://rsbweb.nih.gov/ij/index.html).

## Supporting Information

Figure S1 Codon-optimized hANT4 sequence for yeast expression used in this study.
(DOC)

Table S1 Primer sequences used in this study.
(DOC)
