# Supplementary material for: Functional Expression of Human Adenine Nucleotide Translocase 4 in Saccharomyces Cerevisiae
Source: PLoS One. 2011 Apr 21;6(4):e19250. doi: 10.1371/journal.pone.0019250 (PMC3080916; doi:10.1371/journal.pone.0019250)
Supplement: Figure S1 — Codon-optimized hANT4 sequence for yeast expression used in this study. (DOC) [file pone.0019250.s001.doc]

**Supplemental Figure S1.**

Codon-optimized hANT4 sequence for yeast expression used in this study

ATGCATAGAGAACCTGCCAAAAAAAAGGCCGAAAAGAGATTGTTCGACGCT

M H R E P A K K K A E K R L F D A

TCTTCCTTCGGTAAAGACTTGTTAGCCGGTGGTGTCGCTGCTGCTGTTTCT

S S F G K D L L A G G V A A A V S

AAAACCGCCGTTGCCCCAATTGAAAGAGTCAAATTGTTGTTACAAGTTCAA

K T A V A P I E R V K L L L Q V Q

GCCTCCTCCAAACAAATTTCCCCTGAAGCCAGATATAAGGGTATGGTAGAC

A S S K Q I S P E A R Y K G M V D

TGCTTAGTCAGAATCCCAAGAGAACAAGGTTTCTTCTCCTTCTGGAGAGGT

C L V R I P R E Q G F F S F W R G

AATTTGGCCAATGTCATAAGATATTTCCCAACCCAAGCTTTGAATTTCGCC

N L A N V I R Y F P T Q A L N F A

TTCAAGGACAAGTATAAGCAATTATTCATGTCCGGTGTAAACAAAGAAAAG

F K D K Y K Q L F M S G V N K E K

CAATTCTGGAGATGGTTCTTGGCAAACTTGGCCTCTGGTGGTGCTGCCGGT

Q F W R W F L A N L A S G G A A G

GCTACATCTTTGTGTGTCGTCTATCCATTGGACTTCGCTAGAACAAGATTG

A T S L C V V Y P L D F A R T R L

GGTGTCGACATCGGTAAAGGTCCTGAAGAAAGACAATTCAAGGGTTTGGGT

G V D I G K G P E E R Q F K G L G

GATTGTATCATGAAAATCGCCAAATCCGACGGTATTGCTGGTTTGTATCAA

D C I M K I A K S D G I A G L Y Q

GGTTTCGGTGTTTCCGTTCAAGGTATCATCGTCTATAGAGCCTCATATTTC

G F G V S V Q G I I V Y R A S Y F

GGTGCCTATGACACCGTAAAGGGTTTATTGCCTAAACCAAAAAAAACCCCA

G A Y D T V K G L L P K P K K T P

TTCTTAGTCTCATTCTTTATCGCCCAAGTCGTCACAACATGTTCTGGTATT

F L V S F F I A Q V V T T C S G I

TTATCCTATCCTTTCGATACTGTTAGAAGAAGAATGATGATGCAATCCGGT

L S Y P F D T V R R R M M M Q S G

GAAGCCAAAAGACAATATAAGGGTACTTTGGACTGCTTCGTCAAAATCTAT

E A K R Q Y K G T L D C F V K I Y

CAACACGAAGGTATTAGTTCATTTTTTAGAGGTGCCTTTTCCAACGTCTTG

Q H E G I S S F F R G A F S N V L

AGAGGTACTGGTGGTGCCTTGGTCTTGGTCTTGTATGACAAAATCAAGGAA

R G T G G A L V L V L Y D K I K E

TTCTTCCACATCGATATTGGTGGTAGATAA

F F H I D I G G R *
