# Supplementary material for: Functional Expression of Human Adenine Nucleotide Translocase 4 in Saccharomyces Cerevisiae
Source: PLoS One. 2011 Apr 21;6(4):e19250. doi: 10.1371/journal.pone.0019250 (PMC3080916; doi:10.1371/journal.pone.0019250)
Supplement: Table S1 — Primer sequences used in this study. (DOC) [file pone.0019250.s002.doc]

**SUPPLEMENTAL TABLE S1. Primer sequences used in this study.**

| Primer name | Primer sequence*^a^* | Annealed primer |
| --- | --- | --- |
| F1 | TTTTCACGACAACCCACTCA |  |
| R1 | GGCTATTTGCTTATATGTATG |  |
| F2 | GTCTAATCTGGCTTGATTCTTAATC |  |
| R2 | CACCGGCACAAAGAGTGATA |  |
| U1 | GCAAATAGCCCTGGAACAGCGGATCTGAAT | R1 |
| U2 | GCCAGATTAGACATCATTACGACCGAGATTCCC | F2 |
| HisF | CATCACCATCACTCTTCCAACGCCCAAGTCAAAAC | HisR |
| HisR | GTGATGATGGTGCATGGCTATTTGCTTATATGTATG | HisF |
| yNR | CAATCAAAAAGTTAGATTCCTTC |  |
| yNRhANT1F | GAAGGAATCTAACTTTTTGATTGACTTCCTGGCCGGGGGCGTC | yNR |
| yNRhANT2F | GAAGGAATCTAACTTTTTGATTGACTTCCTGGCAGGTGGAG | yNR |
| yNRhANT3F | GAAGGAATCTAACTTTTTGATTGACTTCTTGGCCGGAGGCATC | yNR |
| yNRhANT4F | GAAGGAATCTAACTTTTTGATTGACTTGTTAGCCGGTGGTGTC | yNR |
| R2hANT1R | GAATCAAGCCAGATTAGACTTAGACATATTTTTTGATCTCATCATAC | F2 |
| R2hANT2R | GAATCAAGCCAGATTAGACTTATGTGTACTTCTTGATTTCATC | F2 |
| R2hANT3R | GAATCAAGCCAGATTAGACTTAGATCACCTTCTTGAGCTCGTCGTA | F2 |
| R2hANT4R | GAATCAAGCCAGATTAGACTTATCTACCACCAATATCGATGTGG | F2 |
| Xho1yNF | AGCTACTCGAGATGCACCATCATCACCATCACTCTTCC |  |
| Nhe1hA4R | ATGGCTAGCTTATCTACCACCAATATCGATGTGG |  |
| Nhe1hA2R | CATGGCTAGCTTA TGTGTACTTCTTGATTTCATC |  |
| V5F | GGTGACGGTGCTGGTTTAGGTAAACCAATTCCAAATCCATTGT  TGGGTTTGGATTCTACTTAAGGCGCGCCACTTCTAAATAAG |  |
| V5R | GTGAGAAAGAATTTAGATTAAGAATCAAGCCAGATTAGACTCGATG  AATTCGAGCTCG |  |
| hANT4V5F | CAAAATCAAGGAATTCTTCCACATCGATATTGGTGGTAGAGGTGAC  GGTGCTGGTTTA |  |
| hANT4F | AGCTTTGAATTTCGCCTTCA |  |
| hANT4R | CCAAGTTTGCCAAGAACCAT |  |
| ALG9F | CACGGATAGTGGCTTTGGTGAACAATTAC |  |
| ALG9R | TATGATTATCTGGCAGCAGGAAAGAACTTGGG |  |

*^a^* Underlined sequence is annealed to indicated primer during fusion PCR
